# Application of Adipose-Tissue Derived Products for Burn Wound Healing

**DOI:** 10.3390/ph16091302

**Published:** 2023-09-14

**Authors:** Hamid Malekzadeh, Zayaan Tirmizi, José A. Arellano, Francesco M. Egro, Asim Ejaz

**Affiliations:** Department of Plastic Surgery, University of Pittsburgh, Pittsburgh, PA 15261, USA

**Keywords:** adipose tissue, stem cells, burns, radiation, exosomes

## Abstract

Burn injuries are a significant global health concern, leading to high morbidity and mortality. Deep burn injuries often result in delayed healing and scar formation, necessitating effective treatment options. Regenerative medicine, particularly cell therapy using adipose-derived stem cells (ASCs), has emerged as a promising approach to improving burn wound healing and reducing scarring. Both in vitro and preclinical studies have demonstrated the efficacy of ASCs and the stromal vascular fraction (SVF) in addressing burn wounds. The application of ASCs for burn healing has been studied in various forms, including autologous or allogeneic cells delivered in suspension or within scaffolds in animal burn models. Additionally, ASC-derived non-cellular components, such as conditioned media or exosomes have shown promise. Injection of ASCs and SVF at burn sites have been demonstrated to enhance wound healing by reducing inflammation and promoting angiogenesis, epithelialization, and granulation tissue formation through their paracrine secretome. This review discusses the applications of adipose tissue derivatives in burn injury treatment, encompassing ASC transplantation, as well as the utilization of non-cellular components utilization for therapeutic benefits. The application of ASCs in burn healing in the future will require addressing donor variability, safety, and efficacy for successful clinical application.

## 1. Introduction

Burns are the fourth most common cause of injury in the world. In 2019, more than nine million burn cases globally were estimated, resulting in 111,000 mortalities (88,000–132,000) [1,2]. Nonfatal burn injuries also often result in severe morbidities including a prolonged hospital stay and physical scarring and disfigurement, influencing mental health and causing a decreased quality of life [3]. Burns commonly result from thermal and radiation sources, while chemical burns, though less frequent, also occur. Although various etiologies may have a distinct pathophysiology, the wound-healing process remains fundamentally similar across different causes. Burns can be classified into different degrees based on the depth of injury: first-degree burns are confined to the epidermis, superficial partial-thickness burns impact the superficial dermis, deep partial-thickness burns extend into the deeper dermis, and full-thickness burns encompass the entire skin thickness, extending to subcutaneous structures [4].

## 2. The Pathophysiology of Burn Wound Healing

**Figure 1:**
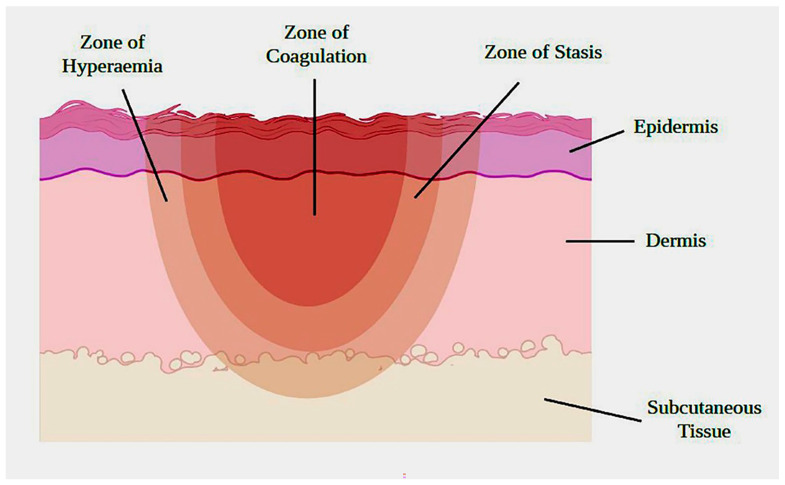
Zones of burn injury.

Acute burn injury triggers the production of reactive oxygen species (ROS) and leads to the denaturation of proteins, oxidative stress, and tissue necrosis [5,6]. After an injury, a burn area includes three zones: the zone of coagulation (central), the zone of stasis (middle), and the zone of hyperemia (outermost) (Figure 1). The zone of coagulation is associated with irreversible damage and vascular destruction, while the zone of hyperemia is the result of inflammatory vasodilation and recovers without any scarring. The zone of stasis, however, shows variable outcomes and may be a potential target for intervention [4,6].

An acute burn injury is usually followed by a robust inflammatory response marked by the recruitment of neutrophils and macrophages into the site of injury. During the inflammatory phase, macrophages and neutrophils serve to remove debris and pathogens and release a variety of growth factors including transforming growth factor-β (TGF-β), vascular endothelial growth factor (VEGF), epidermal growth factor (EGF), and pro-inflammatory cytokines such as IL-1 and IL-8 [4,7].

**Figure 2:**
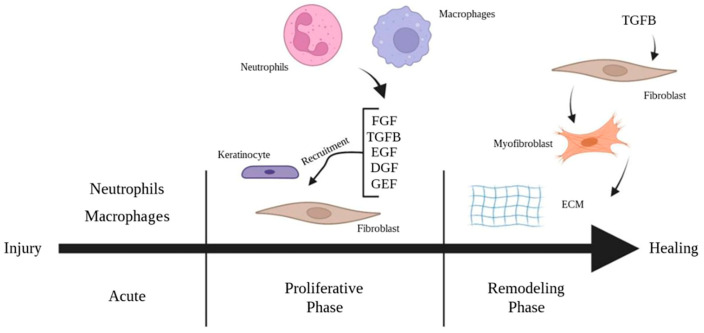
Phases of burn injury.

The proliferative phase follows the initial inflammatory response and is characterized by granulation tissue formation, collagen deposition, epithelialization, and angiogenesis. This phase involves the activation of keratinocytes and fibroblasts via various cytokines and growth factors and migration to the site of injury. Fibroblasts migrate to the edge of the wound in response to the platelet-derived growth factor (PDGF), TGF-β, and fibroblast growth factor (FGF) produced by platelets and macrophages [7,8]. Fibroblasts play a central role in the formation of granulation tissue, as they synthesize and deposit extracellular matrix proteins that provide structural support for its development [9] (Figure 2).

In addition to ECM production, neovascularization plays a critical role in the development of granulation tissue. It is driven by endothelial cell activation by growth factors including VEGF, hepatocyte growth factor (HGF), and FGF. Fibroblasts also contribute to angiogenesis by releasing angiogenic growth factors such as FGF, PDGF, and VEGF while also decreasing matrix metalloproteinase activity. The final phase of wound healing involves the remodeling of the extracellular matrix characterized by the replacement of initial type III collagen with more organized type I collagen and scar contracture by myofibroblasts. 

Re-epithelialization starts at the wound edges early in the proliferative phase as the basal keratinocytes migrate and proliferate across the viable wound bed [10]. The loss of contact inhibition of locomotion and the release of local growth factors including EGF, TGF-α, and keratinocyte growth factors (KGF) signal keratinocyte migration. However, keratinocyte migration from the wound edge is limited to only 1 to 2 cm. In superficial burns, migrating keratinocytes originate not only from the wound edge but also from the skin adnexa and hair follicles. These structures harbor undamaged epithelial cells that play a vital role in replenishing the injured area [8,9,10].

However, in deep partial-thickness and full-thickness burns, adnexal damage and the presence of the burn eschar often disrupt the healing process [11]. In these deeper burns, re-epithelialization is limited to basal keratinocytes at the wound edges [10]. Deeper burns are also often accompanied by a potent inflammatory response that further delays healing and disrupts angiogenesis. This delayed neovascularization plays a significant role in many of the acute and subacute sequelae of thermal injury including the risk of scarring and infection [11]. These burns are often accompanied by impaired re-epithelialization and remodeling phases, leading to the excessive deposition of extracellular collagen and scar formation. Activation of fibrotic pathways through angiotensin II and TGF-beta play an important role in fibrotic scar development [12]. In addition, the transformation of fibroblasts into myofibroblasts plays a pivotal role in ongoing extracellular matrix (ECM) remodeling. This process can lead to excessive wound contraction and disruption to the collagen matrix, ultimately culminating in the development of hypertrophic scars [9].

### 2.1. Current Practice and Limitations

The current standard of treatment for full-thickness wounds involves a combination of surgical intervention, advanced wound dressings, and supportive therapies. Surgical intervention typically involves debridement of the non-viable tissue and multiple skin grafting or flap reconstruction. Advanced wound dressings are utilized to enhance wound healing by maintaining moisture balance, facilitating cellular migration and proliferation, and reducing the risk of infection [10]. However, treatment with skin grafts is limited by the availability of donor sites, graft failure, and scarring and it is usually insufficient in reconstructing skin and soft-tissue defects. Despite notable advancements in burn treatment techniques, including the use of novel skin-grafting methods, tissue-engineered skin substitutes, synthetic dressings, and the topical application of growth factors, challenges persist in the effective regeneration of skin defects and prevention of hypertrophic scarring.

### 2.2. Regenerative Medicine Approaches for Wound Healing

Regenerative medicine has emerged as a promising solution to address the shortcomings of current treatments. By leveraging the body’s natural capacity to repair and regenerate damaged tissues, it offers a potential breakthrough in promoting burn wound healing, minimizing scarring, and restoring normal skin function. There are several approaches and techniques utilized in regenerative therapy for wound healing, including cell-based products, hydrogel, scaffold matrices, and growth factors [13]. The application of stem cells, in particular, has been extensively investigated as a potential strategy to enhance wound healing. The unique regenerative properties displayed by stem cells offer great potential in improving wound-healing outcomes and advancing the field of burn treatment.

Following an injurious insult, endogenous stem cells are activated and migrate to the site of injury, playing a pivotal role in the intricate signaling cascades of wound healing [14,15]. Stem cells exert their beneficial effects through various mechanisms. They exhibit the capacity to differentiate into specific cell types crucial for tissue repair, such as keratinocytes, fibroblasts, and endothelial cells [16]. However, emerging evidence suggests that the primary mechanism by which stem cells enhance the wound-healing process is through paracrine interactions with local and immune cells. Stem cells secrete an array of cytokines and growth factors that modulate the wound microenvironment, thereby mitigating inflammation and promoting angiogenesis, cell proliferation, and extracellular matrix deposition [14,17].

**Figure 3:**
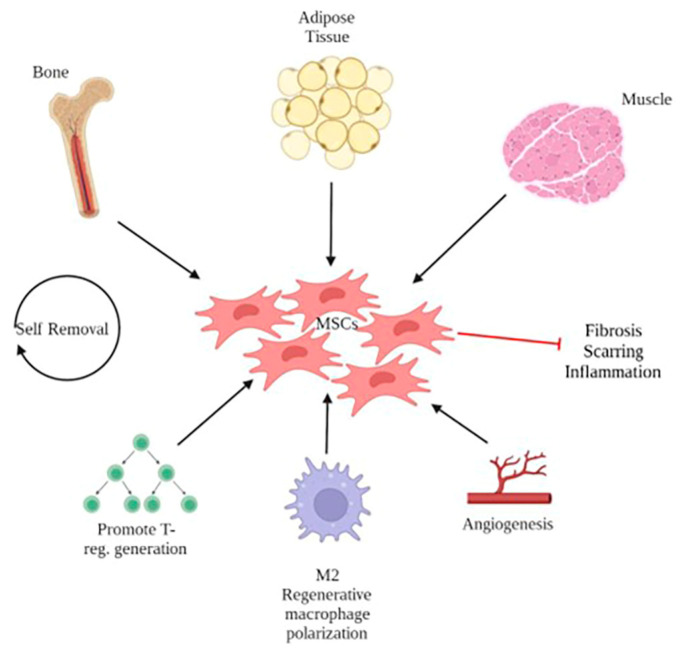
Regenerative therapeutic properties of mesenchymal stem cells.

Mesenchymal stem cells (MSCs) are widely utilized as a choice of cell therapy among researchers due to their multifaceted therapeutic applications (Figure 3). MSCs can be derived from different tissues such as adipose tissue, bone marrow, and umbilical cord. They exhibit immunological inactivity, characterized by low expression of the human leukocyte antigen class I (HLA I) and the absence of HLA class II [18,19]. MSCs have a self-renewal capacity and limited differentiation potential. Notably, they also demonstrate immunomodulatory properties, promoting the generation of T-reg cells and shifting the macrophage phenotype towards an anti-inflammatory M2 state. This phenotypic shift enhances wound healing by stimulating fibroblast proliferation and suppressing inflammation. Additionally, MSCs foster a balanced inflammatory and anti-inflammatory cytokine response, facilitating angiogenesis, reducing fibrosis, and minimizing wound scarring [18].

Conditioned medium from MSCs contains many of the factors involved in wound healing including PDGF, VEGF, FGF, EGF, KGF, and TGF-β [17]. In vitro experiments have demonstrated that treatment with MSC-conditioned medium substantially enhances the migration and proliferation of keratinocytes and fibroblasts [20,21]. Furthermore, the conditioned medium of adipose-derived stem cells (ASCs) upregulates the production of collagen type I and III and fibronectin, suggesting an important role for soluble factors derived from ASCs in the improvement of wound healing [22].

Stem cells exert paracrine effects through diverse signaling pathways including PI3K/AKT, WNT-β-catenin, and TGF-β. The activation of PI3K/AKT expedites re-epithelialization, fibroblast migration, and angiogenesis in wound healing [23,24]. Similarly, the Wnt/β-catenin pathway guides epithelial cell migration and keratinocyte differentiation, while also influencing fibroblast migration [25]. The TGF-β pathway plays a pivotal role in wound healing. Conditioned media and exosomes from MSCs stimulate fibroblast proliferation and migration via TGF-β [19,26]. Dysregulation results in scarring, fibrosis, and collagen irregularities [27]. Collectively, these pathways illuminate stem cells’ therapeutic potential for enhanced wound healing.

### 2.3. Application of Adipose-Tissue–Derived Products in Burn Wounds

**Figure 4:**
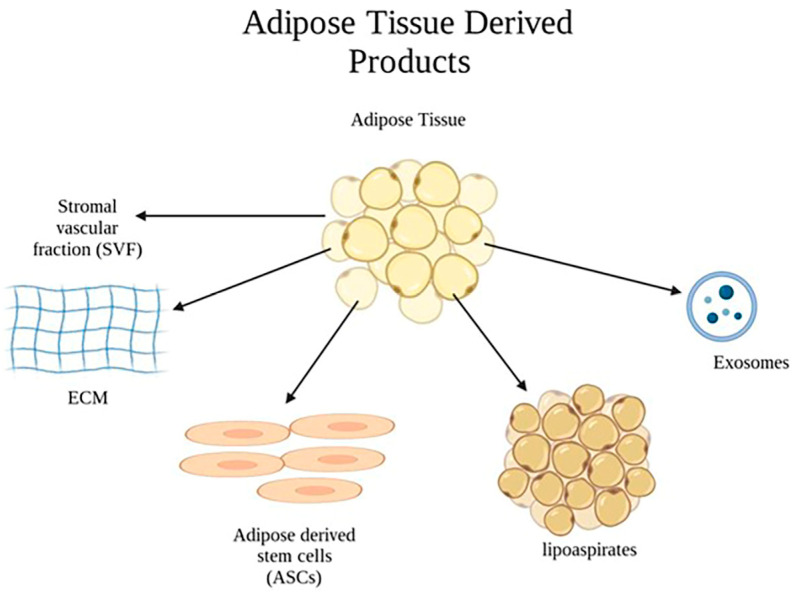
Adipose-tissue-derived products having potential therapeutical applications.

Previous clinical studies have reported that autologous fat grafting may accelerate healing in chronically scarred tissue resulting from chronic wounds and radiation-induced soft tissue injury [28,29]. In the field of burn treatment, fat grafting has shown promising outcomes as an adjuvant therapy by promoting wound healing, reducing the typical healing time, and minimizing the occurrence of hypertrophic scarring [30]. In this context, the utilization of adipose tissue derivatives has gained significant attention. The regenerative properties and immunomodulatory effects of adipose-derived stem cells have sparked great interest in exploring their therapeutic potential for enhancing the wound-healing process and mitigating scar formation. This article aims to provide an in-depth review of the application of adipose tissue derivatives as valuable tools in the treatment of burn wounds, highlighting their impact on improving wound healing and exploring the underlying mechanisms responsible for these beneficial effects (Figure 4) [30,31].

Among the various types of MSCs, ASCs possess several advantages that make them particularly suitable for clinical applications. Adipose tissue provides an abundant and easily accessible source of stem cells, obtained through minimally invasive procedures like liposuction, which result in a low morbidity and a higher yield of ASCs. In comparison to other MSC types, ASCs exhibit a superior proliferative capacity and enhanced differentiation potential into multiple cell lineages involved in wound healing, such as keratinocytes, fibroblasts, and endothelial cells [32,33]. Moreover, ASCs have a low immunogenicity and minimal ethical concerns, making them a viable choice for therapeutic interventions. Overall, the unique attributes and therapeutic potential of ASCs position them as an attractive option for wound healing, offering prospects for enhanced clinical outcomes and accelerated tissue regeneration [32,34].

### 2.4. Adipose-Derived Stem Cells (ASCs)

**Figure 5:**
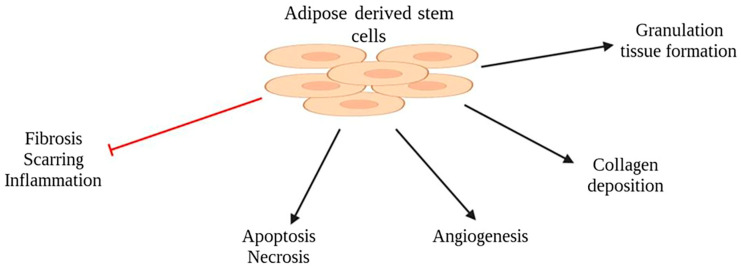
ASCs function in wound healing.

ASCs have become a focus of interest in reconstructive surgery due to their regenerative and immunomodulatory properties (Figure 5). Several animal studies have investigated the impact of ASC treatment on partial- and full-thickness burn injuries and reported accelerated wound healing evidenced by a reduced burn surface area and depth [11,35,36,37,38]. Additionally, these studies have reported a decrease in inflammatory infiltration and levels of inflammatory markers, including myeloperoxidase activity for neutrophils and malondialdehyde levels as an indication of reduced oxidative stress [35,37,39]. Moreover, a decrease in both apoptosis and necrotic areas within the treated wounds has been reported [11,39]. Both human and mouse ASCs have demonstrated the ability to enhance angiogenesis and promote the deposition of collagen type III by the fourteenth day after injury, which corresponds to the granulation tissue formation and the production of the extracellular matrix during the proliferative phase of wound healing [37,38,40]. Furthermore, increased hair follicle growth has been observed during the third and fourth week after injury, indicating the regeneration of skin appendages in addition to accelerated wound healing. Furthermore, studies have shown a decrease in inflammatory infiltration, fibrosis, scar tissue formation, and lymphatic vessels during the maturation/remodeling phase of wound healing [38,40].

The potential benefits of ASCs in the context of radiation-induced burn injuries have also been demonstrated. Huang et al. observed a decreased wound size and increased capillary density at 3 weeks in rats exposed to radiation and treated with ASCs compared to the control group (PBS) [41]. Riccobono et al. demonstrated that autologous ASCs led to improved wound healing and an absence of necrosis compared to controls, while no significant difference was observed with allogeneic ASCs [42]. Furthermore, Wu et al. reported accelerated wound healing with ASCs and adipose-derived products such as centrifugated fat and fragmented adipose connective tissue in a mouse model of radiation-induced skin injury [43].

The efficacy of ASCs at the wound site and their survival have been a point a concern in translational research. Bliley et al. demonstrated the integration of human ASCs within the burn wound of an athymic mouse model three weeks after injection [40]. Autologous and allogeneic ASCs have both been investigated in previous experiments. Notably, autologous and syngeneic ASCs have consistently yielded improved healing in all experiments [11,36]. However, a study by Chang et al. reported no significant difference for allogeneic ASCs compared to the control group, while improved wound healing was observed with autologous ASCs. The authors discussed that this discrepancy in the study of allogeneic ASCs might be attributed to the injection of cell-culture medium in their control group, in contrast to other studies where no treatment served as the control [36]. Other studies have investigated the application of allogeneic ASCs for burn injury in murine models and have reported favorable outcomes on wound healing [37,39,40].

### 2.5. Strategies for Delivering ASCs to Enhance Retention at the Wound Site

The delivery of cells, particularly stem cells, using hydrogels or scaffolds is a prominent tissue-engineering approach aimed at enhancing engraftment and survival rates at the wound site [44]. Scaffolds, typically made of biocompatible materials, provide structural support and mimic the extracellular matrix, facilitating cell adhesion, migration, and proliferation. They offer a framework for cell growth and tissue integration, while also allowing for nutrient and oxygen diffusion to support healing processes [45]. Meanwhile, hydrogels, composed of highly hydrated polymer networks, possess unique properties that make them ideal for burn wound applications. Hydrogels can absorb and retain large amounts of water, maintaining a moist environment that promotes epithelialization and prevents wound desiccation. Additionally, they can encapsulate bioactive molecules such as growth factors, antibiotics, and stem cells, enabling controlled release and targeted therapeutic interventions [46]. Advancements in scaffolds, hydrogels, and tissue-engineered substitutes have shown significant potential in the development of advanced burn dressings and regenerative therapies. These innovative approaches offer promising solutions to address the complex challenges associated with severe burn injuries, ultimately leading to an improved quality of life for burn patients.

Combining ASCs with scaffolds and hydrogels has shown promising results in improving wound healing. Previous studies have demonstrated that ASCs, when combined with these biomaterials, enhanced various aspects of the healing process. The combination of ASCs with hydrogel-based systems (including pullulan–collagen [6], collagen–polyethylene glycol [47], gelatin/microbial transglutaminase [48], hyaluronic acid [49], and dextran/carboxymethyl chitosan [50]) accelerated wound closure, promoted re-epithelization, and facilitated better matrix deposition and scar remodeling [6,47,48,49,50]. Moreover, the combination of ASCs with either scaffolds or hydrogels (including decellularized human amniotic membrane [51,52], poly(3-hydroxybutyrate-co-hydroxyvalerate) [53], and collagen peptide [54,55]) decreased inflammation and fibrosis, improved granulation tissue formation, and increased levels of proangiogenic factors [6,50,51,52,53]. Similarly, in the context of scaffold-based approaches, ASCs contributed to increased hair follicle and sebaceous gland development, more complex collagen deposition, and enhanced angiogenesis [51,52,53].

However, it is important to note that only a few of these studies included a control group of ASCs alone, limiting the ability to attribute the observed results solely to the synergistic effect of biomaterial and stem cells [6,48]. Nevertheless, these findings highlight the potential of combining ASCs with biomaterials to enhance wound-healing outcomes. This combination approach shows promise in modulating critical processes such as cell migration, tissue regeneration, angiogenesis, and extracellular matrix remodeling, ultimately improving the overall healing response.

### 2.6. Stromal Vascular Fraction (SVF)

**Figure 6:**
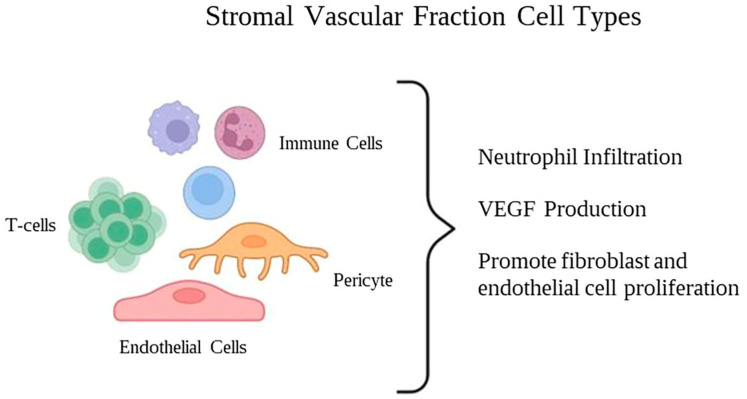
Cellular composition of the adipose tissue stromal vascular fraction.

Th stromal vascular fraction (SVF) is a heterogenous mixture of cells including ASCs, mesenchymal and endothelial progenitor cells, smooth muscle cells, pericytes, fibroblasts, macrophages, and other immune cell subtypes (Figure 6).

The SVF is obtained through mechanical and enzymatic dissociation followed by centrifugation to separate the different cellular components. After processing and administration, the SVF cells can differentiate into different cell types, support neovascularization, replace cells, and repair injured tissue. Hence, the SVF has gained attention in regenerative medicine and has been utilized in tissue engineering and wound-healing research [56,57].

Previous in vivo studies have demonstrated that treatment with freshly isolated SVF leads to reduced neutrophil infiltration and a faster resolution of the inflammatory phase within 7 to 12 days in partial- and full-thickness burns. Intradermal injection and topical spray of SVF have been shown to enhance VEGF levels, wound re-epithelialization, neovascularization, and the proliferation of fibroblasts and endothelial cells within the wound bed and the zone of stasis [58,59]. In a swine model, the utilization of SVF loaded in a collagen scaffold resulted in an increased thickness of granulation tissue, collagen deposition, and neovascularization when compared to the control scaffold group [60].

Studies have demonstrated that conditioned media obtained from both fresh and frozen human SVF can stimulate the migration and proliferation of keratinocytes and fibroblasts. Additionally, after 24 h of culture, both fresh and frozen SVF exhibit high expression levels of growth factors associated with regeneration, such as EGF, FGF2, HGF, and VEGF. In a murine burn model, treatment with human SVF has been shown to reduce the wound surface area and enhance skin regeneration by increasing the production of type I collagen and promoting neovascularization in the granulation tissue. Interestingly, an analysis of the tissue did not reveal any transplanted cells, indicating that wound healing was facilitated by a paracrine effect rather than direct cell replacement [61].

### 2.7. Cell Derived Exosomes

Exosomes, which are nanoscale extracellular vesicles secreted by most cell types, mediate intercellular communication through paracrine pathways. In the last decade, exosomes have been widely studied to develop novel therapeutic strategies to address challenging clinical issues, particularly chronic wounds. They exhibit remarkable biocompatibility and immune stability, rendering them a promising approach for enhancing the healing of chronic wounds over other therapeutic modalities such as stem cell transplantation [62]. Recent studies have demonstrated the regenerative potential of mesenchymal stem cell (MSC)-derived exosomes in key processes involved in wound healing including cell proliferation, cell migration, and neovascularization [63].

**Figure 7:**
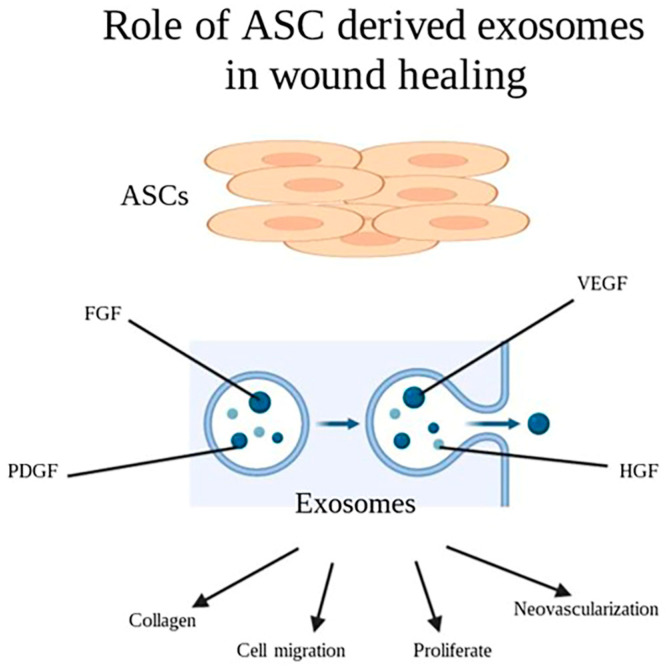
Role of ASCs derived exosomes in wound healing.

Recent studies investigating the roles of ASC-derived exosomes in wound healing have suggested exosomes are internalized by keratinocytes and fibroblasts and induce cell migration, proliferation, and collagen deposition in a dose-dependent response [64,65]. Exosomes were shown to include growth factors important to the wound-healing process, such as VEGF-A, FGF-2, HGF, and PDGF in in vitro studies (Figure 7) [66]. In a mouse incisional wound model, ASC-derived exosomes enhanced collagen deposition and wound healing and reduced scar-tissue formation. This effect was accompanied by the increased expression of genes involved in cell adhesion genes (N-cadherin), cell-cycle regulation (cyclin-1, PCNA), and collagen production (collagen I, III) [65]. ASC-derived exosomes hold promise as a novel therapeutic approach for skin soft tissue repair, but further animal studies are required.

### 2.8. From Bench to Bedside

The translation of pre-clinical studies using stem cells into human studies for burn healing is a relatively recent development. Most of the clinical studies have primarily investigated the use of bone marrow MSCs (BM-MSCs) for the treatment of burn wounds. The first clinical application of stem cells for acute burn injury treatment was reported in 2005. In this case, a patient with an extensive skin burn underwent skin grafting with allogeneic BM-MSCs transplantation onto the deep thermal burn surface. The treatment resulted in accelerated wound healing characterized by increased re-epithelialization, neovascularization, and reduced scarring [67].

Two additional case reports have described the application of autologous and cadaveric BM-MSCs in conjunction with skin grafting for severe burn cases and reported improved wound-healing outcomes, including enhanced re-epithelialization, neovascularization, and reduced contraction [68,69]. Another study reported on two severe burn cases in which allogeneic BM-MSCs were cultured on a collagen scaffold artificial dermis sheet, resulting in accelerated wound healing, increased re-epithelialization, and decreased scarring [70]. Moreover, a prospective case–control study demonstrated the efficacy of both bone-marrow- and umbilical-cord-derived MSCs in improving wound healing and reducing complications. These benefits included reduced scarring and contracture formation [71]. The application of ASCs was reported in a case of refractory forearm contracture in the context of a severe childhood burn and multiple reconstructive surgeries. Following the application of an SVF seeded in a collagen scaffold, the patient was able to regain most of her range of motion at the 5-year follow-up [72].

The application of adipose tissue derivatives has also been studied for the treatment of chronic wounds following radiation therapy. In a clinical study, 20 patients with chronic lesions after radiation therapy underwent purified autologous lipoaspirate transplantation. The treatment resulted in improved or remitted symptoms in all evaluated patients, even those with initial irreversible functional damage [73]. Akita et al. treated a patient with an intractable sacrococcygeal wound, 40 years after radiation therapy, by debriding the wound and injecting isolated autologous ASCs and lipoaspirate. They reported uneventful healing by Day 82, with no recurrence [74]. In a case report by Iddins et al., a patient developed an ionizing radiation-induced wound on the thumb which recurred despite medical treatments. The debridement of the affected area, followed by an SVF injection into the wound bed, led to successful healing without recurrence during the one-year follow-up period [75].

### 2.9. Limitations and Future Directions

The use of adipose-derived products, particularly adipose stem cells (ASCs), for burn healing, offers substantial therapeutic potential. However, it is crucial to recognize and address the limitations and challenges encountered in this rapidly advancing field.

One of the limitations for the usage of ASCs in regenerative medicine is the variability in the ASCs sourced from donor-related factors including differences in ages, body mass indexes (BMIs), and the presence of chronic diseases. The immunomodulatory capabilities of ASCs tend to decline with donor age. An elevated BMI negatively affects ASC proliferation and differentiation potentials, making it a critical criterion for donor selection in clinical applications [76]. Chronic diseases, such as diabetes, can also affect ASC potency for wound healing. However, studies have shown accelerated wound healing with autologous ASCs in diabetic swine models and diabetic patients [77,78,79]. These factors make standardization challenging and necessitate rigorous donor-selection processes.

Determining the most effective route for ASC administration (e.g., local injection or systemic infusion) remains an ongoing challenge, with each approach having distinct advantages and disadvantages. Ensuring precise localization of ASCs within the burn wound is crucial for optimal outcomes.

Furthermore, the long-term safety and the potential for tumorigenicity of ASCs must be thoroughly investigated before widespread clinical implementation. Reports of adverse outcomes including neoplastic lesion development have necessitated strict FDA regulations on purified stem-cell therapy for patient safety [80]. The shift towards SVF application led to the prohibition of specific preparation methods involving the enzymatic disruption and ultrasonic cavitation of fat tissue. Currently, only minimal manipulation fat grafting complies with FDA guidelines [81,82]. Although existing research is promising, comprehensive preclinical and clinical trials are essential to elucidate these concerns.

The current literature on the application of ASCs for burn wound treatment is limited to experimental and small-scale clinical studies. Although several clinical studies on adipose stem-cell potential to treat burn injury have been registered on clinicaltrials.gov (accessed on 1 March 2023) the details for their recruitment status and results are not known (Table 1). Overall, more clinical studies on the effects of adipose tissue derivatives including ASCs and SVF are needed, as these have already shown promising results in preclinical burn studies and some clinical wound-healing trials.

In conclusion, addressing the limitations in ASC application for burn wound healing necessitates comprehensive research, adherence to regulatory guidelines, and robust clinical exploration. By conducting further clinical trials, we can better understand the safety and efficacy of ASCs and related adipose tissue derivatives, paving the way for more effective and ethical treatments for burn patients in the future.

## Figures and Tables

**Table 1:** Clinical studies on adipose stem-cell potential to treat burn injury.

	Condition or Disease	Intervention/ Treatment	Phase	Sponsor:	Location	First Posted/ Last Update	Recruitment Status	Results
NCT02394873	Deep Second-degree burn	ALLO-ASC-DFU (a hydrogel containing allogenic ASCs)	Phase 1	Antero gen Co., Ltd.	Seoul, Korea	March 2015/ December 2015	Completed	Not available
NCT03183622	Deep Second-degree burn	ALLO-ASC-DFU (a hydrogel containing allogenic ASCs)	Phase 1	Antero gen Co., Ltd.	Seoul, Korea	June 2017/ January 2018	Completed	Not available
NCT02619851	Deep Second-degree burn	ALLO-ASC-DFU (a hydrogel containing allogenic ASCs)	Phase 2	Antero gen Co., Ltd.	Seoul, Korea	December 2015/ July 2021	Unknown	Not available
NCT03183648	Deep Second-degree burn	ALLO-ASC-DFU (a hydrogel containing allogenic ASCs)	Phase 2	Antero gen Co., Ltd.	Seoul, Korea	June 2017/ July 2021	Unknown	Not available
NCT03113747	Second- or Third-degree Burns	Allogeneic ASCs	Phase 1 Phase 2	A A Partners, LLC	Kyiv, Ukraine	April 2017/ April 2017	Unknown	Not available
NCT03686449	Full-Thickness	ASC-Keratinocyte Suspension	-	-	Cairo, Egypt	September 2018/May 2020	Unknown	Not available

## Data Availability

Data sharing is not applicable.
